# Scrollable Display of Radial Sectional Computed Tomography Images for Complex Mitral Valve Anatomy

**DOI:** 10.1016/j.atssr.2023.09.006

**Published:** 2023-09-28

**Authors:** Takeichiro Nakane, Akihiro Isotani, Aya Miyazaki, Akira Marui, Nobuhisa Ohno

**Affiliations:** 1 Department of Cardiovascular Surgery, Kokura Memorial Hospital, Kitakyushu City, Japan; 2 Department of Cardiology, Kokura Memorial Hospital, Kitakyushu City, Japan; 3 Department of Radiology, Kokura Memorial Hospital, Kitakyushu City, Japan

## Abstract

**Background:**

Advances in computed tomography (CT) technology have increased its utility in the diagnosis of mitral regurgitation (MR). Herein, we report on a unique dual-panel display for high-resolution valve images of radial multiplanar reconstruction named mitral valve–radial sectional view (MV-RSV) and a case of mitral valve repair for MR that demonstrates the usefulness of this display method.

**Methods:**

Whole-heart images from contrast-enhanced CT were acquired with electrocardiographic gating and processed to reconstruct 3-dimensional and cross-sectional images. The new display consisted of the selection panel and cross section panel, the latter showing the desired radial section selected by scrolling to present the morphologic features of the mitral valve leaflets and subvalvular structures. The patient was a 63-year-old man with MR who underwent valve repair. In addition to echocardiography, the mitral valve was evaluated preoperatively and postoperatively by CT using MV-RSV.

**Results:**

Preoperative MV-RSV revealed P2 prolapse where severe MR with chordal rupture was assessed by transesophageal echocardiography. After the mitral valve repair with chordal plasty and ring annuloplasty, MV-RSV clarified the gap between leaflets at the P2-P3 indentation, which was associated with mild residual leakage.

**Conclusions:**

The new scrollable CT display is a simple and convenient method to show the structure of lesions that cause MR and is useful for developing treatment strategies and postoperative evaluation.

## Visual Abstract

**Figure:**
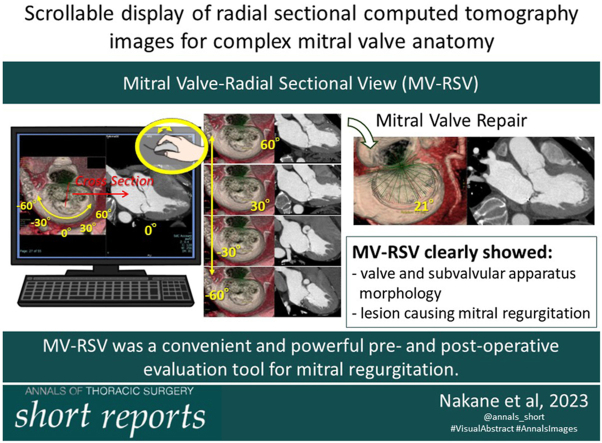

**In Short**

▪ A unique dual-panel display for radial sectional computed tomography images of the mitral valve, mitral valve–radial sectional view (MV-RSV), showed the morphologic features of corresponding anterior and posterior leaflets at the selected position conveniently by scrolling.
▪ MV-RSV clarified the lesion that caused mitral regurgitation and is useful for preoperative and postoperative evaluation of mitral valve repair.
▪ MV-RSV is a convenient and powerful evaluation tool that complements echocardiography.

Whereas echocardiography is the primary method for diagnosis of mitral regurgitation (MR), advances in computed tomography (CT) technology have made it possible to reconstruct accurate images, increasing the importance of this tool in the evaluation and treatment of MR.^1^^,^^2^ The usefulness of a large quantity of data obtained by CT can be greatly enhanced through proper methods to display reconstructed images. We devised a unique dual-panel display for high-resolution images of radial section multiplanar reconstruction (MPR) named mitral valve–radial sectional view (MV-RSV), which visualized the structure of corresponding anterior and posterior leaflets at the selected position by scrolling. Here, we report a case of mitral valve repair for MR to demonstrate the usefulness of MV-RSV.

## Patients and Methods

### CT Protocol

In our institute, cardiac CT scanning to evaluate the mitral valve is performed with Revolution CT (GE Healthcare), a 256-detector row multidetector CT scanner. A bolus of nonionic iodinated contrast material (Iopamiron 370 [Bayer HealthCare] or Iopamidol 370 [Fuji Pharma]) is injected at a rate of 24 mgI/kg per second for 13 seconds, followed by a 30-mL saline flush. One-beat comprehensive heart data are then acquired with electrocardiographic gating. Scan parameters are as follows: tube voltage, 100 kVp; automatic tube current modulation system (Smart mA) with an optimal range of 100 to1080 mA; rotation time, 0.28 sec/rot; noise index, 27; field of view, 200 mm with 512 × 512 matrix size; section thickness, 0.625 mm; collimation, 160 mm. After transfer of multiphase source data to the Advantage Workstation VolumeShare 7XT (version 4.7; GE Healthcare), postprocessing is performed for image reconstruction by artificial intelligence–based deep learning image reconstruction technology and SnapShot Freeze 2.0 (SSF2; GE Healthcare), an automated motion correction algorithm.^3^ SSF2 uses information from phases adjacent to the target cardiac phase in a single cardiac cycle and corrects the motion of structures within the entire heart, including the coronary artery, myocardium, valves, and great vessels. It reduces the blurring of artifacts due to cardiac motion by 6 times that of conventional methods, with 24 milliseconds of effective temporal resolution at a 0.28-second rotation speed.^3^^,^^4^ GE's multimaterial artifact reduction, a cone beam spectral artifacts correction algorithm, is applied for beam hardening.^5^

### Mitral Valve Image Reconstruction and Display, MV-RSV

**Figure 1:**
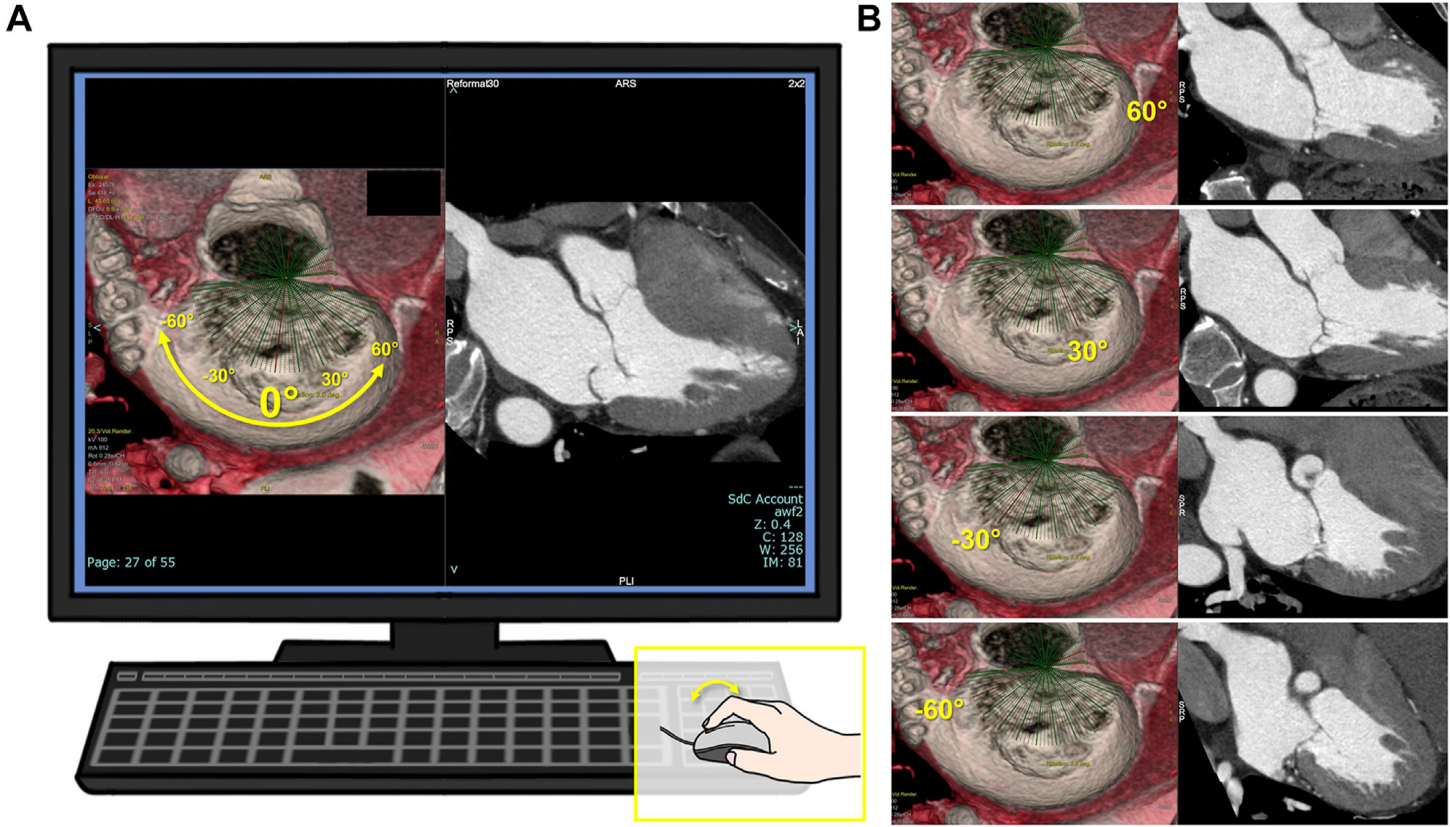
Preoperative computed tomography findings in mitral valve–radial sectional view. (A) Illustration of the scrollable computed tomography display, mitral valve–radial sectional view, with the 0° image of the current case. The left panel shows en face 3-dimensional mitral valve volume rendering image with radial green lines every 3°. The right panel shows the cross-sectional multiplanar reconstruction image at the red line (0°), which can be selected by scrolling. The image describes the prolapse of the P2. (B) Cross-sectional images when scrolling and selecting 60°, 30°, −30°, and −60°, respectively, showing appropriate coaptation of anterior and posterior at each site.

Our new display, MV-RSV, consists of 2 panels—the selection panel and cross section panel (Figure 1A). From the collected data, a short-axis cross section of the mitral valve at the end-systolic phase is reconstructed. The short-axis section is slabbed at 30 to 40 mm and displayed using volume rendering (VR). The VR is then adjusted to the direction of the en face view from the left atrium, which resembles that of 3-dimensional transesophageal echocardiography, called the surgeon’s view.^6^ This image is displayed on the left of Figure 1A with radial green lines. In general, lines perpendicular to the coaptation line of the mitral valve cross near the middle of the anterior annulus (Supplemental Figure). Therefore, we settled it as a “center,” and the line connecting the center and the middle of the posterior annulus is defined as the 0° line, from which a line is drawn to −90° and +90° every 3° around the center. The cross section panel on the right of Figure 1A shows an MPR image corresponding to the radial line colored in red in the left panel, which is nearly perpendicular to the coaptation line. The VR and MPR images are saved in pairs, and the user can select the view continuously on the selection panel using the scroll wheel (Figure 1B; Video 1).

### Case

**Figure 2:**
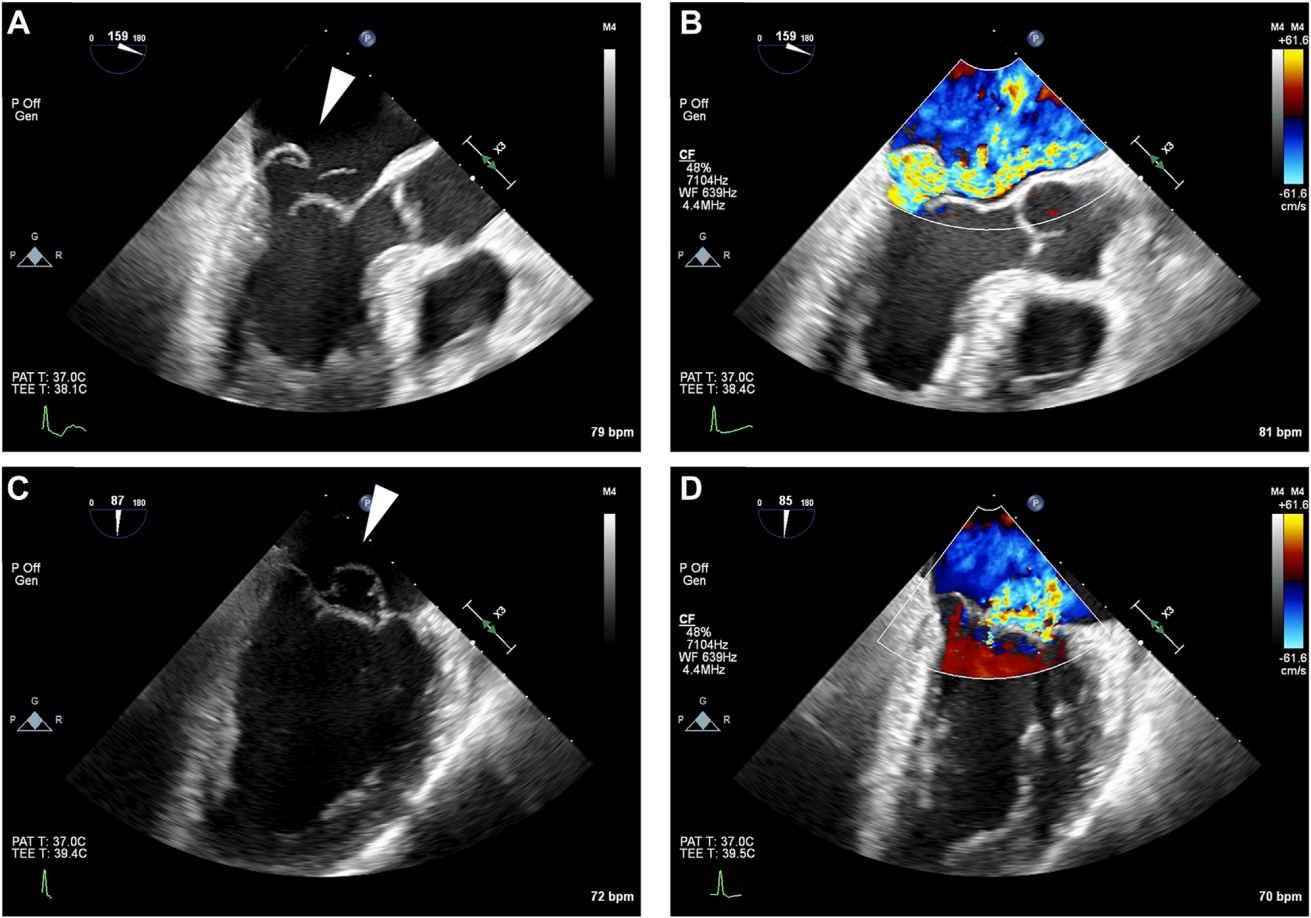
Preoperative transesophageal echocardiography findings. (A, B) Midesophageal aortic valve long-axis view. (C, D) Midesophageal mitral commissural view. (B, D) Echocardiography color Doppler images. The arrowheads in (A) and (C) indicate P2 prolapse with chordal rupture, and severe mitral regurgitation is assessed by color Doppler in (B) and (D).

The patient was a 63-year-old man with MR. Transesophageal echocardiography showed severe MR due to P2 prolapse, which was associated with chordae rupture and annular dilation (Figure 2). Mild tricuspid regurgitation with annular dilation was also observed, and the tricuspid regurgitation pressure gradient was increased to 35 mm Hg. Minimally invasive mitral valve repair and tricuspid annuloplasty through right mini-thoracotomy were indicated. Written informed consent for publication was obtained from the patient.

## Results

### Operative Findings of the Mitral Valve

**Figure 3:**
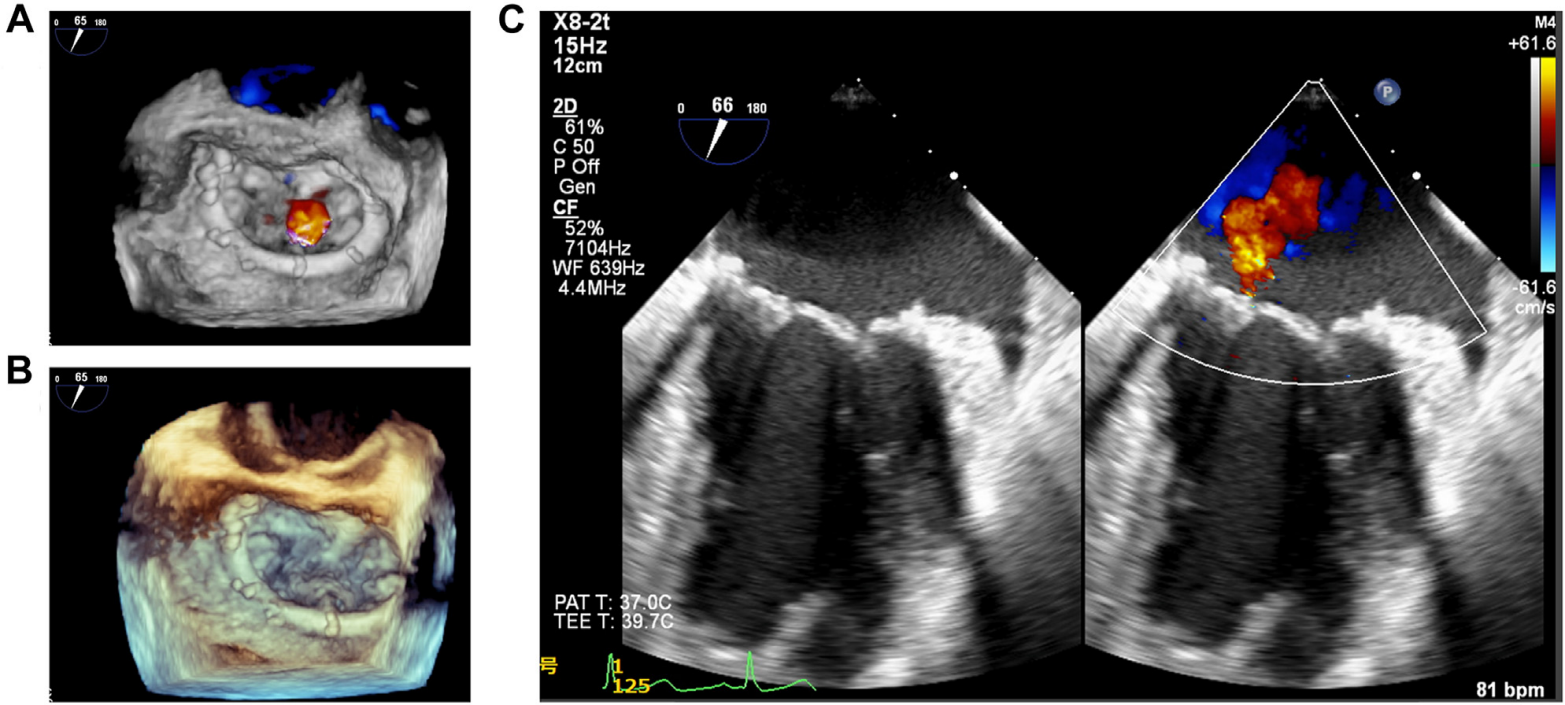
Intraoperative transesophageal echocardiography findings after mitral valve repair. (A, B) Three-dimensional constructed mitral valve images. (C) Midesophageal mitral commissural view. After the repair with chordal plasty and ring annuloplasty, the mitral valve shape is corrected, but a central jet corresponding to mild regurgitation is revealed at the indentation between P2 and P3.

The P2 was entirely prolapsed with 4 torn chordae tendineae. In addition, the P2 had degenerated and thickened, and the edge of the contralateral anterior scallop was also thickened. Two pairs of 4-0 expanded polytetrafluoroethylene suture artificial neochordae were implanted from the anterior and posterior papillary muscles into the P2 lesion, and annuloplasty with a 38-mm Physio Flex annuloplasty ring (Edwards Lifesciences) was performed. The leakage test showed that the prolapse was corrected, and a sufficient coaptation margin of the leaflets was confirmed. However, a small amount of leakage was noted from the indentation between P2 and P3, which was attributed to the sclerosing degeneration of the lesion. An additional suture to the indentation was considered, but P3 was normal, whereas P2 was sclerosed. We were concerned that the suture would deform the P3 and exacerbate the leakage. Because there was also degeneration of the contralateral anterior scallop, we determined that complete control of the leakage would be difficult and decided to terminate the repair. Transesophageal echocardiography after aortic declamping showed residual mild MR from the indentation site (Figure 3).

### CT Findings in MV-RSV

#### Preoperative Evaluation

The posterior leaflet of the mitral valve prolapsed from −27° to 21°, with the greatest deviation at 0°. The anterior to posterior valve was 35.4 mm in diameter, and the anterior leaflet was 28.0 mm in length (Figure 1, Video 1).

#### Postoperative Evaluation

**Figure 4:**
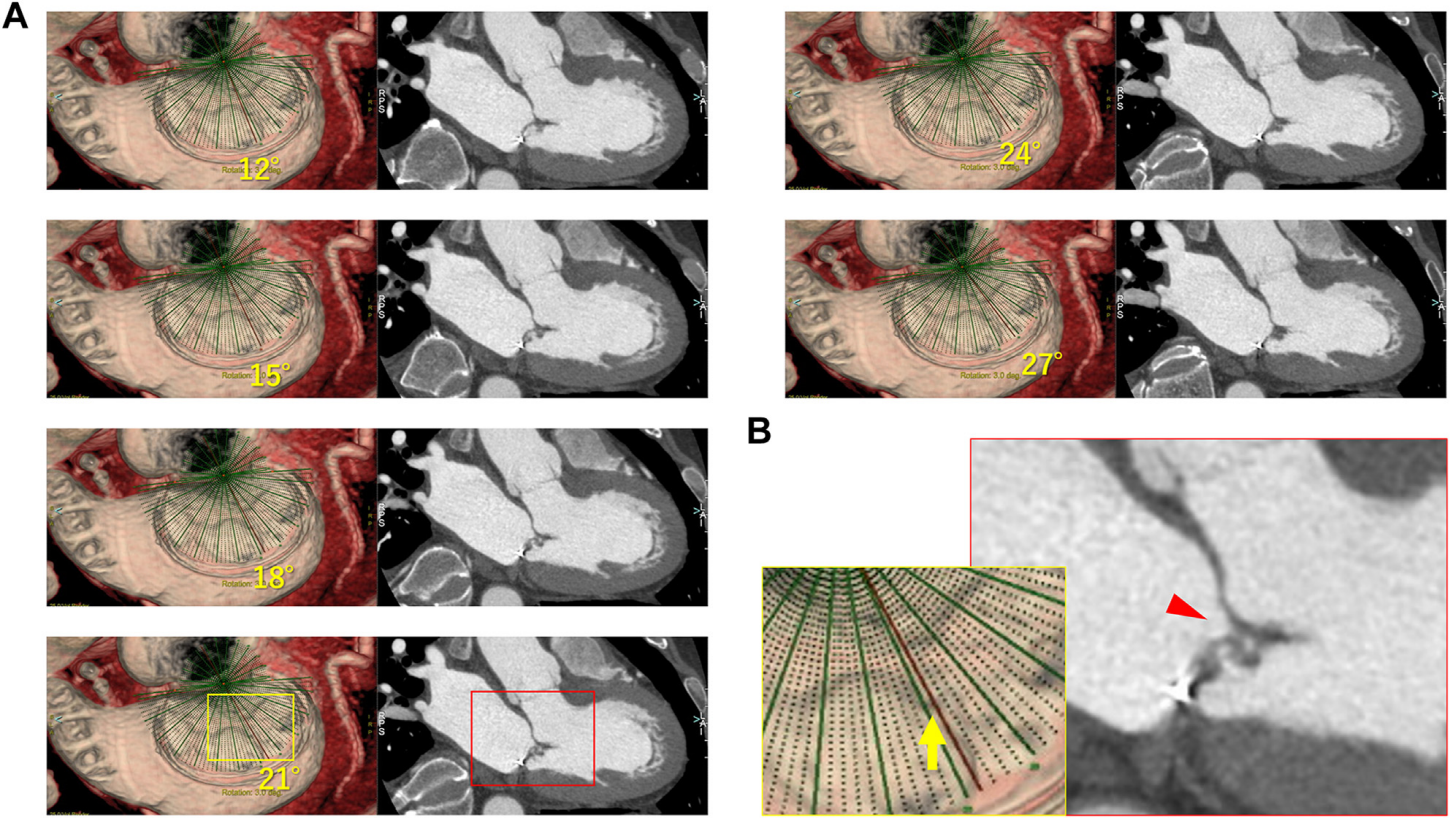
Postoperative computed tomography findings in mitral valve–radial sectional view. (A) Continuous images at 12° to 27° every 3°. (B) Enlarged views of the areas marked with yellow and red squares in the image at 21° in (A). The yellow arrow indicates the indentation between P2 and P3, and the red arrowhead clarifies a gap between the anterior posterior leaflets.

Postoperative transthoracic echocardiography indicated mild MR as well as intraoperative evaluation. Although CT showed that the height of the posterior leaflet was almost corrected with sufficient coaptation to the anterior leaflet, the image at 21° revealed that the anterior cusp did not fit with the posterior one at the indentation between P2 and P3, creating a gap between leaflets through which the regurgitation was observed in the intraoperative leakage test (Figure 4, Video 2). Additional procedures, such as pulling the posterior leaflet down a bit more toward the left ventricle with the neochordae or supplementing the leakage site with autologous pericardium, may have been effective.

## Comment

We introduced a unique display method for mitral valve CT images. CT technology has progressed to reconstruct high-resolution images of the mitral valve at any cross section at any given timing. Thus, we believed it necessary to establish a standardized method of displaying images to use large CT data easily and effectively that satisfied the following requirements: the display is a familiar method compatible with existing imaging examinations, shows reasonable views in which it was easy to identify where the section was cut, and is not laborious to reconstruct. MV-RSV was then developed, having been inspired by the surgeon's view 3-dimensional image and its cross-sectional images in transesophageal echocardiography. It displayed an overall view of the mitral valve side-by-side with a cross-sectional view nearly perpendicular to the coaptation line, and the section could be scrolled and moved continuously along the coaptation line. Whereas it is difficult for anyone aside from the echographer performing the examination to fully understand the positioning of the displayed view in echography, MV-RSV made the location clear and provided images without being limited by the skill of echographers or the anatomy of the patient. Because Resolution CT achieves a spatial resolution of 0.23 mm, it described the continuity from the valve leaflet to the papillary muscle more clearly than echocardiography. Blooming artifacts induced by calcification or prostheses can be obstacles; the root causes of the artifacts are partial volume averaging, motion, and beam hardening.^7^ Partial volume effects could be reduced by reducing slice thickness (0.625 mm), motion artifacts by SSF2, and beam hardening by multimaterial artifact reduction, allowing the reconstruction of diagnosable images even in cases with valvular calcification or after valve repair with annuloplasty rings.^7^ Regarding heart rate and arrhythmia, it is reported that motion artifacts are corrected by SSF2 more effectively at higher heart rates, and the accuracy of images processed with SSF2 is not affected by heart rate or arrhythmias.^4^^,^^8^^,^^9^ Thus, MV-RSV is a powerful evaluation tool to complement echocardiography in understanding the structure of the mitral valve and subvalvular apparatus. Furthermore, its benefit could be greater in cases in which transesophageal examination is intolerable because of congestive heart failure or other reasons.^10^ In our hospital, MV-RSV images are obtained for nearly all patients with MR requiring surgery to decide treatment strategies at our heart team meetings. When MR remained in postoperative transthoracic echocardiography, morphologic problems could be examined with MV-RSV without the need for invasive transesophageal echocardiography. Therefore, this method was also helpful for postoperative evaluation, providing valuable information for reviewing the performed procedure and consequently for surgical education. Transcatheter mitral valve repair has recently been developed for treatment of MR.^10^ MV-RSV is also applicable in the perioperative evaluation of transcatheter procedures, and its usefulness is certain to increase in the future. Potential barriers to the implementation of this protocol include the risk of complications related to injection of contrast material, such as allergy and renal impairment.

The scrollable CT display, MV-RSV, was shown to be a simple and convenient method that takes advantage of the characteristics of CT to show the structure of lesions that cause MR and was useful for developing treatment strategies and postoperative evaluation.

## Figures and Tables

**Supplemental Figure 1:**
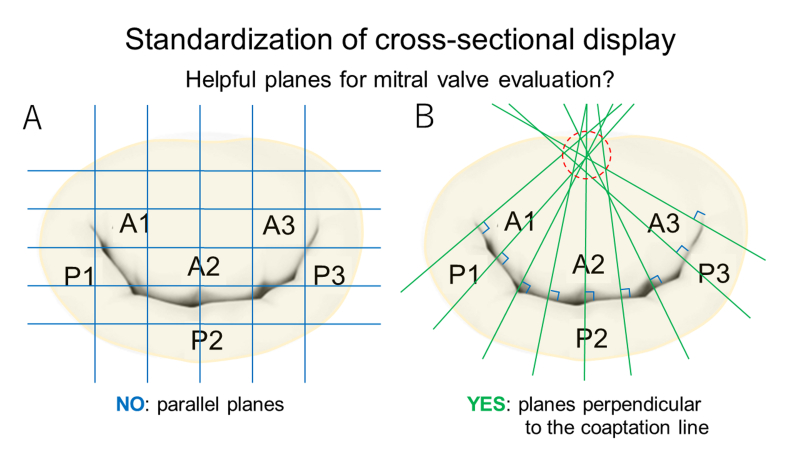


## Supplementary Data

Legend for Supplemental Figure 1 and Videos 1 and 2
