# Supplementary material for: Scrollable Display of Radial Sectional Computed Tomography Images for Complex Mitral Valve Anatomy
Source: Ann Thorac Surg Short Rep. 2023 Sep 28;2(1):52–7. doi: 10.1016/j.atssr.2023.09.006 (PMC11708432; doi:10.1016/j.atssr.2023.09.006)
Supplement: Legend for Supplemental Figure 1 and Videos 1 and 2 [file mmc1.docx]

**Supplemental Information**

**Supplemental Figure 1.** Illustration of the mitral valve: consideration of the useful method of displaying cross sectional CT images for the assessment of mitral regurgitation.

(A) The grid-like cross section shown by the blue line is not very useful. (B) The cross section we want is perpendicular to the coaptation line. The green line indicates a line perpendicular to each site at the coaptation line. The area where the green lines cross is circled by the red dashed line and is generally located at the center of the anterior annulus.

**Supplemental Video 1.** A video of preoperative Mitral Valve-Radial Sectional View scrolling.

**Supplemental Video 2.** A video of postoperative Mitral Valve-Radial Sectional View scrolling.
